# A Heterogeneous Mixture of F-Series Prostaglandins Promotes Sperm Guidance in the *Caenorhabditis elegans* Reproductive Tract

**DOI:** 10.1371/journal.pgen.1003271

**Published:** 2013-01-31

**Authors:** Hieu D. Hoang, Jeevan K. Prasain, Dixon Dorand, Michael A. Miller

**Affiliations:** 1 Department of Cell Biology, Developmental and Integrative Biology, University of Alabama at Birmingham, Birmingham, Alabama, United States of America; 2 Department of Pharmacology and Toxicology, University of Alabama at Birmingham, Birmingham, Alabama, United States of America; University of California San Francisco, United States of America

## Abstract

The mechanisms that guide motile sperm through the female reproductive tract to oocytes are not well understood. We have shown that *Caenorhabditis elegans* oocytes synthesize sperm guiding F-series prostaglandins from polyunsaturated fatty acid (PUFA) precursors provided in yolk lipoprotein complexes. Here we use genetics and electrospray ionization tandem mass spectrometry to partially delineate F-series prostaglandin metabolism pathways. We show that omega-6 and omega-3 PUFAs, including arachidonic and eicosapentaenoic acids, are converted into more than 10 structurally related F-series prostaglandins, which function collectively and largely redundantly to promote sperm guidance. Disruption of omega-3 PUFA synthesis triggers compensatory up-regulation of prostaglandins derived from omega-6 PUFAs. *C. elegans* F-series prostaglandin synthesis involves biochemical mechanisms distinct from those in mammalian cyclooxygenase-dependent pathways, yet PGF_2α_ stereoisomers are still synthesized. A comparison of F-series prostaglandins in *C. elegans* and mouse tissues reveals shared features. Finally, we show that a conserved cytochrome P450 enzyme, whose human homolog is implicated in Bietti's Crystalline Dystrophy, negatively regulates prostaglandin synthesis. These results support the model that multiple cyclooxygenase-independent prostaglandins function together to promote sperm motility important for fertilization. This cyclooxygenase-independent pathway for F-series synthesis may be conserved.

## Author Summary

A fundamental question in cell and developmental biology is how motile cells find their target destinations. One of the most important cell targeting mechanisms involves the sperm and oocyte, which unite during fertilization to produce the next generation of offspring. We have been using the nematode *C. elegans* to delineate these mechanisms. Our prior studies have shown that oocytes secrete F-series prostaglandins that stimulate sperm motility. Prostaglandins are widespread signaling molecules derived from polyunsaturated fatty acids or PUFAs. Mammals are not capable of synthesizing PUFAs and must receive them in the diet. *C. elegans* was not thought to synthesize prostaglandins because the genome lacks cyclooxygenases, enzymes that catalyze the rate-limiting step in mammalian prostaglandin synthesis. Here we show that *C. elegans* oocytes synthesize a heterogenous mixture of structurally related F-series prostaglandins derived from different PUFA classes, including the enantiomer of PGF_2α_. These prostaglandins function collectively and redundantly to guide sperm to the fertilization site. Our results indicate that F-series prostaglandins can be synthesized independent of cyclooxygenase enzymes. This novel pathway may be evolutionarily conserved. Evidence is emerging that prostaglandins regulate sperm motility in the female reproductive tract of humans.

## Introduction

The union of oocyte and sperm, known as fertilization, is paramount to the survival of animal species [1]–[4]. Oocytes tend to be large stationary cells filled with nutrients to support early embryonic development. Sperm, on the other hand, are smaller mobile cells capable of sensing their environment and responding with changes in motility. Sperm have a relatively simple job—find an oocyte and provide it with a second set of chromosomes. While much has been learned about sperm motility in aquatic species [5], [6], the mechanisms that guide sperm to oocytes in internally fertilizing animals are less well understood. These guidance mechanisms are likely crucial for reproductive success [7], [8]. Evidence is emerging that lipid signaling molecules, such as steroids and prostaglandins are important regulators of sperm motility in the human female reproductive tract [9]–[13].

**Figure 1:**
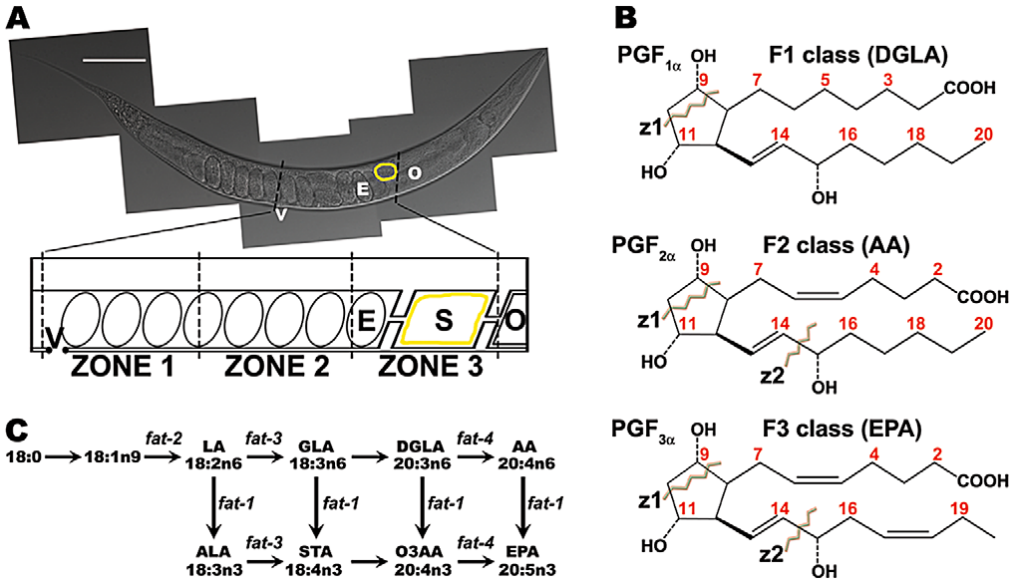
*C. elegans* gonad, F-series prostaglandin structures, and PUFA metabolism pathways. (A) The *C. elegans* hermaphrodite reproductive tract. A differential interference contrast (DIC) image of an adult hermaphrodite is shown above with a diagram of half the uterus below. Males inject sperm through the vulva (V) into the uterus. Sperm become motile by extending a pseudopod and crawl around fertilized embryos (E) toward the spermatheca (S, yellow outline). Oocytes (O) enter the spermatheca during oocyte maturation and ovulation. The uterus is divided into 3 zones for quantification of sperm distribution. Scale bar, 0.1 mm. (B) Human F-series prostaglandin structures showing carbon numbering and important energy-induced cleavage sites characteristic of F-series prostaglandins. z1 cleavage causes loss of 44 Da from the parent ion and z2 cleavage, together with methyl terminal loss, generates the product ion *m/z 193*. Prostaglandin precursors for each class are shown in parentheses. (C) Diagram of *C. elegans* PUFA synthesis pathways. **Δ**9 and **Δ**12 desaturase activities generate substrates for the n3 desaturase FAT-1, the **Δ**6 desaturase FAT-3, and the **Δ**5 desaturase FAT-4 [31]. Fatty acids are abbreviated as in 20:4n6, which has 20 carbons with four double bonds, the first occurring at the n6 position. LA, linoleic acid; ALA, linolenic acid; GLA, **γ**-linolenic acid; STA, stearidonic acid; DGLA, dihomo-**γ**-linolenic acid; O3AA, omega-3 arachidonic acid; AA, arachidonic acid; EPA, eicosapentaenoic acid.

*Caenorhabditis elegans* is a powerful model to study sperm behavior and fertilization [2], [4], [14]–[16]. Its transparent epidermis allows visualization of sperm distribution and motility in live wild-type and mutant animals (Figure 1A) [17]–[19]. Sperm velocity, directional velocity toward eggs, and reversal frequency within the reproductive tract can be quantified from time-lapse videos [18], [20]. Genetic and biochemical analyses have shown that oocytes synthesize F-series prostaglandins (PGs) from polyunsaturated fatty acids (PUFAs) provided in yolk lipoprotein complexes [18], [20], [21]. PUFAs are fatty acids with two or more double bonds, which are essential for prostaglandin cyclopentane ring formation (Figure S1) [22]. Mutations in PUFA biosynthesis enzymes and predicted prostaglandin synthases cause nonautonomous defects in sperm motility, resulting in inefficient targeting to the site of fertilization and reduced reproductive output [18], [20]. Introducing nanomolar concentrations of F-series prostaglandins into the uteri of prostaglandin-deficient mutants has an immediate effect on sperm motility [20]. Liquid chromatography coupled to electrospray ionization tandem mass spectrometry (LC-MS/MS) has identified multiple F-series prostaglandins in *C. elegans* extracts [20], [21]. The synthesis of CePGF2, an analog of PGF_2∝_, is dependent on 20-carbon PUFA synthesis and transport to oocytes [20]. However, the extent to which specific loss of CePGF2 or other prostaglandins affects sperm guidance is not clear. It is also not clear whether *C. elegans* F-series prostaglandins are derived from a single or multiple PUFA precursors.

Mammals lack the desaturase enzymes necessary to convert monounsaturated fatty acids into PUFAs [23], [24]. A key enzyme in mammalian prostaglandin synthesis is cyclooxygenase or PG G/H synthase, which converts PUFAs into the intermediate PGH (Figure S1) [25]. There are three prostaglandin classes that are synthesized from distinct PUFA precursors [26]–[28] (Figure 1B). The F1 class is derived from dihomo-gamma-linolenic acid (DGLA), a 20-carbon PUFA with three double bonds in the carbon backbone. DGLA is also called 20:3n6, where the third double bond occurs at the n6 or omega-6 position. The F2 class is derived from arachidonic acid (AA, 20:4n6; Figure S1B) and the F3 class is derived from the omega-3 PUFA eicosapentaenoic acid (EPA, 20:5n3). PGF_2∝_ is a member of the F2 class synthesized from AA by the sequential action of cyclooxygenase and PGF synthase (Figure S1) [22], [25]. 11β-PGF_2∝_ and 9β-PGF_2∝_ stereoisomers are formed from PGD_2_ and PGE_2_ intermediates, respectively [29]. In addition, a wide array of F-series prostaglandin isomers is formed via cyclooxygenase-independent mechanisms mediated by free radical-initiated peroxidation [30]. In most cases, the biological activities of F-series prostaglandins are not known.

The *C. elegans* PUFA biosynthetic pathways have been delineated (Figure 1C) [31], [32]. Four fatty acid desaturases encoded by *fat* genes convert monounsaturated fatty acids into omega-3 and omega-6 PUFAs. Here we use mutations in *fat* genes, together with LC-MS/MS, to delineate F-series prostaglandin synthesis pathways. We show that multiple F-series prostaglandins, whose formation in large part requires an adult germ line, act collectively to promote sperm guidance. F2 class prostaglandin synthesis does not involve H_2_, D_2_, and E_2_ intermediates that are characteristic of mammalian cyclooxygenase-dependent pathways (Figure S1). We identify a conserved cytochrome P450 enzyme that functions to inhibit prostaglandin metabolism, possibly by diverting their precursors into an alternative pathway(s). Our results support the model that *C. elegans* oocytes secrete a blend of F-series prostaglandins for guiding sperm to the fertilization site.

## Results

### PUFAs act collectively to promote sperm guidance

**Figure 2:**
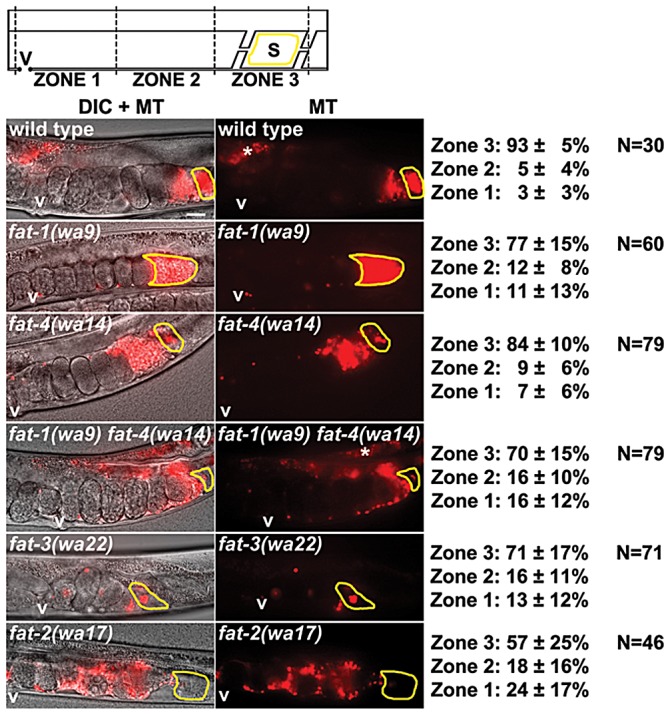
Sperm guidance in wild-type and *fat* mutant hermaphrodites. Uterine sperm distribution 1 hr after mating wild-type or mutant hermaphrodites to wild-type males. A representative image is shown to the left and average zone distribution ± standard deviation is to the right. The spermatheca (S) is outlined in yellow. Asterisks indicate nonspecific gut autoflourescence. *fat-2* and *fat-3* mutants have reduced distance from vulva to spermatheca relative to the wild type, a feature that may influence distribution values. MT, MitoTracker channel; N, number of gonads scored; V, vulva. Scale bar, 20 µm. See Materials and Methods for zone definitions.

To explore the F-series prostaglandin biosynthetic mechanisms important for sperm guidance, we examined *fat* mutants deficient in the synthesis of specific PUFAs classes (Figure 1C). Two quantitative assays were used to evaluate sperm guidance. In both assays, wild-type males are stained with MitoTracker CMXRos dye, which brightly labels sperm [18]. Stained males are mated to unstained mutant or control hermaphrodites and separated. The first assay measures sperm distribution within the uterus one hour after mating, using three defined zones (Figure 1A). In controls, over 90% of sperm move around developing embryos to the fertilization site, called the spermatheca in an hour (Figure 2). The second assay uses time-lapse imaging to directly measure sperm velocity, directional velocity, and reversal frequency within the uterus immediately after mating. Sperm in control uteri move with an average velocity of 8.6 microns per minute, rarely reversing direction. Average directional velocity toward the spermatheca is less than average velocity as sperm migrate around embryos blocking their path (Table 1, line 1).

**Table 1:** Sperm motility values in wild-type and mutant hermaphrodite uteri.

Description	Average Velocity (µm/min)	Average Directional Velocity (µm/min)	Reversal Frequency (rev/hr)	N
1. Wild type*	8.62±0.60	4.28±0.78	1.8	88
2. *fat-1(wa9)*	7.97±0.51	2.50±0.70	9.3	42
3. *fat-4(wa14)*	6.50±0.33	2.08±0.54	5.9	60
4. *fat-1(wa9) fat-4(wa14)*	5.68±0.35	2.74±0.48	5.7	55
5. *fat-3(wa22)* *	3.84±0.31	2.16±0.42	2.5	49
6. *fat-2(wa17)* *	3.85±0.22	0.47±0.30	8.1	94
7. *rme-2(b1008)* **	3.68±0.28	0.45±0.32	9.5	75
8. *glp-4(bn2)* **	3.43±0.25	−0.23±0.25	16.2	79
9. *cyp-31A2(tm2711)*	7.63±0.84	2.04±1.07	3.5	26

The average velocity, average directional velocity toward the spermatheca, and average reversal frequency were determined for wild-type sperm within the uterus (Zone 2) of wild-type and mutant hermaphrodites. Value ± standard error of the mean is shown.

* Includes new data and published data from Kubagawa et al (2006) [18].

** Published data from Kubagawa et al (2006) for reference [18].

First, wild-type males were mated to *fat-1(wa9)* and *fat-4(wa14)* mutant hermaphrodites. *fat-1(wa9)* mutants have undetectable EPA (20:5n3) and omega-3 AA (20:4n3), but have elevated AA (20:4n6) levels relative to the wild type [32], [33]. On the other hand, *fat-4(wa14)* mutants have undetectable EPA and AA, but have elevated omega-3 AA levels [32], [33]. Minor sperm motility defects are evident from sperm distribution and time-lapse imaging assays, but a large percentage of sperm are still able to target the spermatheca (Figure 2 and Table 1, lines 2 and 3). These data raise the possibility that DGLA, which is still synthesized in both *fat-1* and *fat-4* mutants, is the key precursor for sperm guiding prostaglandins.

To test whether or not DGLA is essential, we compared sperm guidance values in *fat-3(wa22)* mutants to *fat-1(wa9) fat-4(wa14)* double mutants. *fat-3(wa22)* mutants contain abundant 18:3n3 (linolenic acid, ALA), but trace amounts of 20:3n3 and undetectable DGLA (20:3n6), AA, omega-3 AA, and EPA [32]. On the other hand, *fat-1(wa9) fat-4(wa14)* double mutants contain abundant DGLA with undetectable AA, omega-3 AA, and EPA [32], [33]. Surprisingly, the sperm guidance values in both mutant strains were similar, albeit significantly less than controls (Figure 2 and Table 1, lines 1, 4, and 5; *P*<0.001). Sperm in both strains have similar directional velocity, although there are differences in velocity and reversal frequency (Table 1, lines 4 and 5). These data indicate that DGLA is not essential for sperm guidance, but it may have a modulatory or partially redundant role.

Finally, we measured sperm guidance in the healthiest 1–2 day old adult *fat-2(wa17)* mutants, avoiding those mutants with considerable pleiotropy. *fat-2* encodes a Δ12 fatty-acyl desaturase that converts monounsaturated fatty acids into PUFAs. *fat-2(wa17)* mutants contain reduced levels of all PUFAs, although approximately 5% Δ12 desaturase activity persists in this strain that is sufficient for prostaglandin synthesis [20], [32]. The sperm guidance defects in *fat-2* mutants are more severe than *fat-3(wa22)* mutants and *fat-1(wa9) fat-4(wa14)* double mutants (Figure 2 and Table 1, lines 4, 5, and 6). Furthermore, the defects are similar to *rme-2(b1008)* mutants, which fail to transport PUFAs to oocytes and *glp-4(bn2)* mutants, which lack oocytes (Table 1, lines 6, 7, and 8) [18], [34], [35]. The trend from *fat* mutant data is that sperm guidance defects inversely correlate with total PUFA content. We conclude that loss of single PUFA classes does not have major effects on sperm guidance. Sperm guidance defects depend on the extent to which PUFA classes are collectively eliminated.

### F-series prostaglandins are synthesized from distinct PUFA pools

**Figure 3:**
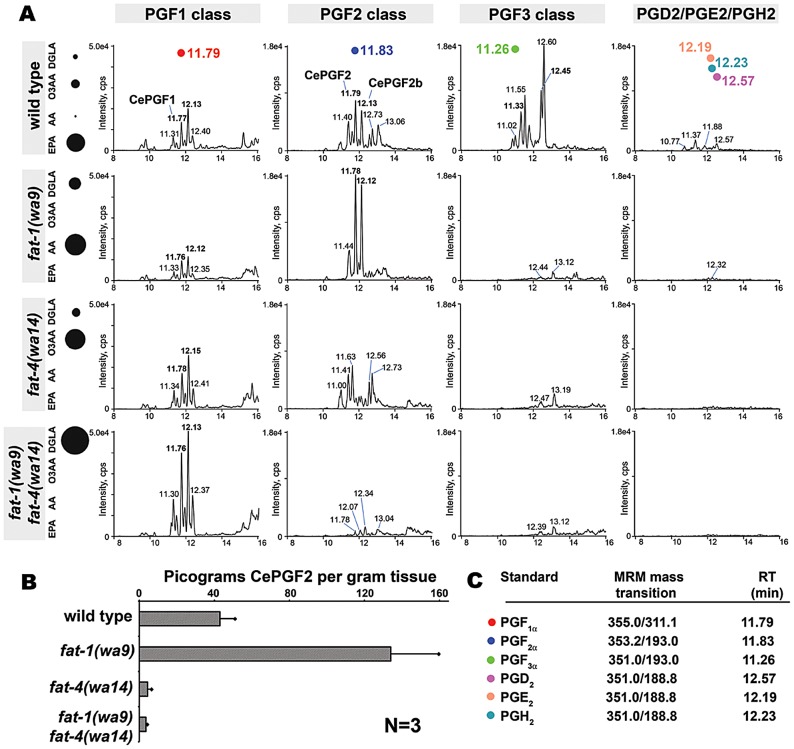
Prostaglandins in wild-type and *fat* mutant extracts. (A) MRM chromatograms of wild-type and *fat* mutant extracts. Liquid chromatography retention time (min) is shown on the X-axis and for selected prostaglandin isomers. Bolded retention times indicate prostaglandin isomers shown in Figure 4. Prostaglandin standard retention times are shown at the top. The dot diagram to the left indicates relative PUFA levels in the corresponding strains [33]. DGLA, dihomo-**γ**-linolenic acid; O3AA, omega-3 arachidonic acid; AA, arachidonic acid; EPA, eicosapentaenoic acid; cps, counts per second. (B) CePGF2 quantification in wild-type and *fat* mutant extracts. Error bars are SD. (C) Summary of MRM mass transitions and retention times for chemically synthesized standards. RT, retention time.

**Figure 4:**
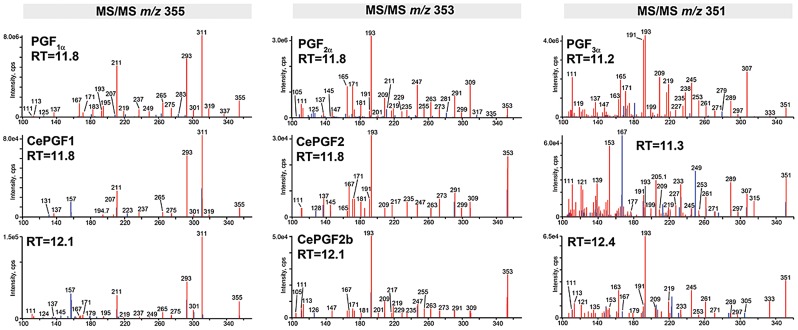
Collision induced decomposition patterns of selected *C. elegans* and human prostaglandins. LC-MS/MS of authentic standards (top) compared to selected *C. elegans* prostaglandins highlighted in Figure 3A. Red color indicates ions shared by the standard and an unknown prostaglandin. Blue color indicates ions that are not shared. *m/z* is on the X-axis. *C. elegans* MS/MS data are from *fat-1(wa9) fat-4(wa14)* mutant extracts (F1 class), *fat-1(wa9)* extracts (F2 class), and wild-type extracts (F3 class). RT, retention time.

A collective requirement for PUFA synthesis could have multiple interpretations. One model is that F-series prostaglandins are synthesized from multiple PUFA classes. A non-mutually exclusive alternative is that worms compensate for loss of a PUFA class by up-regulating prostaglandin synthesis from another class. In both models, prostaglandins from different PUFAs must have largely redundant functions. These (and additional) models were tested through LC-MS/MS analyses of wild-type and *fat* mutant lipid extracts. Mammals synthesize F1, F2, and F3 class prostaglandins from DGLA, AA, and EPA, respectively (Figure 1B and Figure S1). To test whether wild-type worm extracts contained these classes, we used LC-MS/MS operated in multiple reaction monitoring (MRM) mode. In MRM, the parent ion to decomposition (product) ion mass transition is specified, providing excellent specificity and sensitivity. The F1 prostaglandins are detected with mass transition *m/z* 355/311, the F2 class with transition *m/z* 353/193, and the F3 class with transition *m/z* 351/193 or 351/191 [36]–[38]. We found that wild-type extracts contained multiple isomers of each class (Figure 3A). Comparing the collision-induced decomposition (CID or MS/MS) patterns of these prostaglandins to their corresponding chemically synthesized PGF_1α_, PGF_2α_, or PGF_3α_ standards shows extensive similarities (Figure 4, Table 2, and Table 3). Key shared decomposition features include the loss of 44 Da (-C_2_H_4_O) from the parent ion followed by two 18 Da losses (−2× H_2_O), and the presence of the *m/z* 193 ion in the F2 and F3 series (Figure 1B) [36].

**Table 2:** LC-MS/MS data summary for chemically synthesized F-series prostaglandin standards.

Name	RT (min)*	[M-H]^−^ *m/z*	Key product ions in CID (MS/MS)
PGD_2_	12.56	351	315, 271, 233, 203, 189
PGE_2_	12.23	351	333, 315, 271, 235, 189, 175, 109
PGH_2_	12.23	351	333, 315, 271, 235, 217, 189, 175, 113, 109
PGF_1α_	11.83*	355	337, 319, 311, 301, 293, 275, 265, 249, 237, 211, 195
8-iso PGF_1α_	11.34	355	337, 319, 311, 293, 275, 265, 249, 237, 219, 211, 183
9β-PGF_1α_	11.36	355	337, 319, 311, 301, 293, 275, 265, 237, 211,183, 167
8-iso 9β-PGF_1α_	11.44	355	337, 319, 311, 293, 275, 265, 219, 211, 183
9β, 11β-PGF_1α_	11.99	355	337, 319, 311, 301, 293, 275, 265, 237, 219, 211, 183
PGF_2α_	11.73*	353	335, 317, 309, 291, 273, 263, 247, 209, 193, 171, 165
ent-PGF_2α_	11.71	353	335, 317, 309, 291, 273, 247, 209, 193, 191, 171, 165
11β PGF_2α_	11.48	353	335, 317, 309, 291, 273, 247, 209, 193, 173, 165, 111
15(R)-PGF_2α_	11.89	353	335, 317, 309, 291, 273, 247, 209, 193, 191, 171, 165
8-iso PGF_2α_	11.31	353	335, 307, 309, 291, 273, 247, 209, 193, 181, 171, 165
5-trans PGF_2α_	11.60	353	335, 317, 309, 291, 273, 247, 209, 193, 171, 165, 111
8-iso 15(R)-PGF_2α_	11.39	353	335, 317, 309, 291, 273, 263, 247, 209, 193, 171, 165
9β-PGF_2α_	11.22	353	335, 317, 309, 291, 273, 255, 247, 193, 173, 171, 165
PGF_3α_	11.26	351	333, 315, 307, 289, 271, 245, 219, 209, 193, 191, 165
8-iso PGF_3α_	10.83	351	333, 315, 307, 289, 271, 245, 219, 209, 193, 191, 171
2,3-Dinor-11β-PGF_2α_	10.67	325	261, 245, 227, 219, 173, 163, 153, 145, 137, 113, 107
19(R)-hydroxy PGF_2α_	9.19	369	351, 333, 325, 315, 307, 263, 235, 209, 193, 171, 165
20-hydroxy PGF_2α_	9.13	369	351, 333, 325, 315, 307, 263, 209, 193, 181, 171, 165

Retention time (RT), parent ion mass ([M-H]^−^), and key product ion masses are shown for prostaglandin (PG) standards.

* Isomers within each prostaglandin class (i.e. PGF_2α_ isomers) were run together and RTs are directly comparable. PGF_1_ and PGF_2_ classes were run on different days and a slight RT shift is observed. For example, the RTs for PGF_1α_ and PGF_2α_ are indistinguishable when run together.

**Table 3:** LC-MS/MS data for selected *C. elegans* F-series prostaglandins.

Name	RT (min)	[M-H]^−^ m/z	Key product ions in CID (MS/MS)
Class 1	11.24	355	319, 311, 301, 293, 275, 265, 249, 219, 211, 179, 157
CePGF1*	11.70	355	319, 311, 301, 293, 275, 265, 237, 223, 211, 195, 157
Class 1	11.85	355	311, 301, 293, 237, 195, 193, 183, 179, 157, 153
Class 1*	12.08	355	311, 301, 293, 275, 265, 211, 195, 167, 157
Class 1	12.31	355	337, 319, 311, 293, 275, 265, 187, 179, 157, 153
CePGF2*	11.73	353	309, 291, 273, 263, 247, 209, 193, 171, 167, 137
CePGF2b*	12.09	353	309, 291, 273, 263, 255, 247, 219, 209, 193, 171, 113
Class 3*	11.34	351	315, 307, 289, 275, 249, 205, 193, 191, 167, 153, 139
Class 3	11.90	351	307, 289, 263, 249, 193, 191, 185, 147, 137, 115, 109
Class 3*	12.51	351	333, 289, 271, 261, 245, 223, 193, 191, 163
Class 3	12.62	351	289, 271, 245, 223, 219, 201, 193, 183, 149, 113, 107

Retention time (RT), parent ion mass ([M-H]^−^), and key product ion masses are shown. CID data are from wild-type, *fat-1(wa9)* mutant, or *fat-1(wa9) fat-4(wa14)* double mutant extracts. Prostaglandins derived from omega-3 AA are not shown.

* MS/MS data are shown in Figure 4.

One of the two most abundant worm F1 isomers (CePGF1) has a chromatography retention time (RT) that is indistinguishable from PGF_1α_ (Figure 3). Their CID patterns are nearly identical, with the exception of minor ions at *m/z* 157 and 223 (Figure 4). We have previously shown that CePGF2 and PGF_2α_ have indistinguishable chromatography RTs and nearly identical CID patterns [20]. In contrast, the PGF_3α_ RT is several seconds shorter than the closest worm F3 class prostaglandin at RT = 11.33 (Figure 3). While the CID patterns of PGF_3α_ and this prostaglandin are similar, there are more differences than in F1 and F2 classes (Figure 4). One key difference is the abundance of product ion *m/z* 193, which is thought to derive from C14–C15 cleavage and methyl terminus side chain loss (Figure 1B) [36]. This product ion is less abundant in the closest eluting worm F3 prostaglandin compared to PGF_3α_. Another difference is the product ion *m/z* 167, which is not found in PGF_3α_. Therefore, *C. elegans* F3 class prostaglandins are likely to have a significant structural difference relative to PGF_3α_. These data indicate that worms synthesize an array of F1, F2, and F3 class prostaglandins, including CePGF1 and CePGF2.

Next, we examined F-series prostaglandins in *fat-1(wa9)* mutants, *fat-4(wa14)* mutants, and *fat-1(wa9) fat-4(wa14)* double mutants. There are several important features of the data. First, all three mutant strains lack EPA and F3 class prostaglandins (Figure 3A). Second, *fat-1(wa9)* mutants with elevated AA levels have elevated CePGF2 and a second PGF_2α_ analog, called CePGF2b (Figure 3A–3B). Both CePGF2 and CePGF2b are absent in *fat-4(wa14)* mutants and *fat-1(wa9) fat-4(wa14)* double mutants that lack AA (Figure 3A–3B). Third, *fat-4* mutants with elevated omega-3 AA levels have elevated levels of several F2 class isomers (Figure 3A). These prostaglandins are also found in wild-type extracts at lower abundance. Finally, *fat-1(wa9) fat-4(wa14)* double mutants with elevated DGLA have elevated F1 class prostaglandins, including CePGF1 (Figure 3A). Taken together, these data strongly support the model that *C. elegans* extracts contain F1, F2, and F3 class prostaglandins derived from 20∶3, 20∶4, and 20∶5 PUFA precursors, respectively. We conclude that prostaglandin synthesis is regulated in part by precursor abundance.

*fat-3(wa22)* mutants and *fat-1(wa9) fat-4(wa14)* double mutants have similar sperm guidance defects, yet different PUFA content. We previously showed that *fat-3* is required for CePGF2 synthesis, but F1 and F3 class members were not analyzed [20]. A re-examination of *fat-3(wa22)* mutant extracts using LC-MS/MS shows that CePGF1, CePGF2, CePGF2b, and all F3 class prostaglandins are largely absent in *fat-3* mutants (Figure S2A). However, several potential F-series prostaglandins are found, perhaps derived from trace levels of 20:3n3 found in *fat-3(wa22)* extracts [39]. Overall, the total 20-carbon prostaglandin level in *fat-3* mutants is lower than the level in *fat-1 fat-4* double mutants. As *fat-3* mutants have approximately 12-fold higher 18:3n3 (ALA) than the wild type [39], we hypothesized that 18:3n3 might serve as a substrate for prostaglandin synthesis. The 18-carbon PGF_2α_ metabolite 2,3-Dinor 11β PGF_2α_ has a chromatography RT of 10.7 min, so we predicted a prostaglandin derived from 18:3n3 might have a similar RT (Table 3). An 18:3n3 F-series prostaglandin should have parent ion mass *m/z* 327, which is 28 Da lighter (-C_2_H_4_) than PGF_1α_. LC-MS/MS identified a putative F-series prostaglandin at RT = 10.9 min with considerable similarity to PGF_1α_, including loss of 44 Da from the parent ion followed by two 18 Da losses (Figure S2B). MRM using mass transition *m/z* 327/283 shows that this compound and at least one other compound are strongly up-regulated in *fat-3* mutants relative to the wild type (Figure S2C). These data add further support to the model that multiple precursors can generate F-series prostaglandins and precursor abundance influences synthesis.

### F-series prostaglandins have redundant functions in sperm guidance

**Figure 5:**
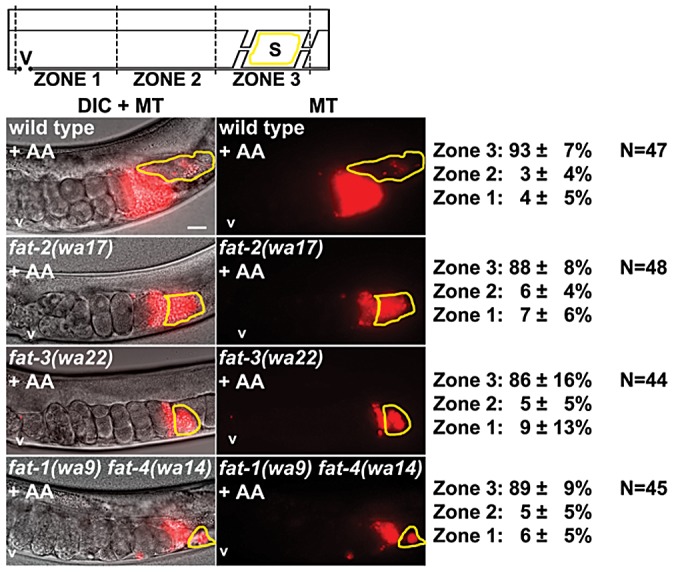
Effect of AA supplementation on sperm guidance in wild-type and *fat* mutant hermaphrodites. Sperm distribution 1 hr after mating AA supplemented wild-type or mutant hermaphrodites to non-supplemented wild-type males. A representative image is shown to the left and average zone distribution ± standard deviation is to the right. See Figure 2 for sperm distribution in non-supplemented *fat* mutants. The spermatheca (S) is outlined in yellow. V, vulva; MT, MitoTracker channel; N, number of gonads scored. Scale bar, 20 µm.

**Figure 6:**
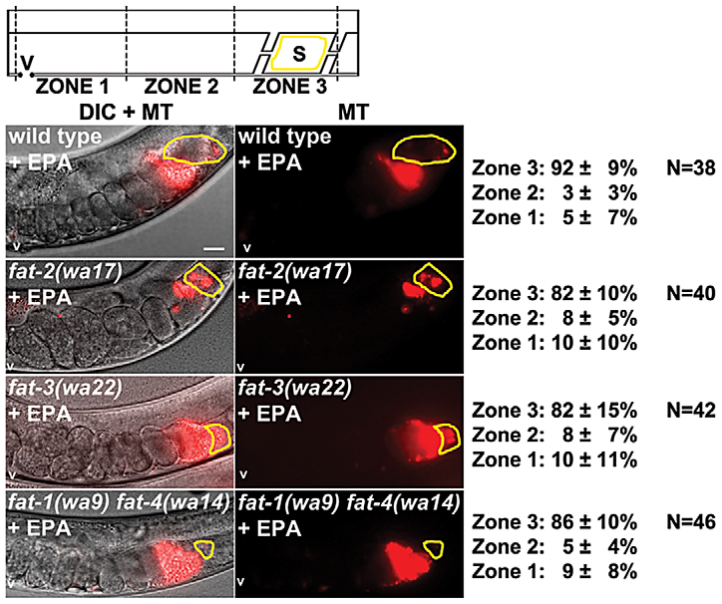
Effect of EPA supplementation on sperm guidance in wild-type and *fat* mutant hermaphrodites. Sperm distribution 1 hr after mating EPA supplemented wild-type or mutant hermaphrodites to non-supplemented wild-type males. A representative image is shown to the left and average zone distribution ± standard deviation is to the right. See Figure 2 for sperm distribution in non-supplemented *fat* mutants. The spermatheca (S) is outlined in yellow. V, vulva; MT, MitoTracker channel; N, number of gonads scored. Scale bar, 20 µm.

The current data provide evidence that F-series prostaglandins have redundant functions. Consistent with this possibility, microinjecting nanomolar concentrations of PGF_2α_ or PGF_3α_ into the uteri of *fat-2(wa17)* or *rme-2(b1008)* mutants has an immediate and similar effect on sperm velocity [20]. To further test this model, we supplemented the diets of wild-type, *fat-3(wa22)* mutant, and *fat-1(wa9) fat-4(wa14)* double mutant hermaphrodites with AA or EPA. Whereas *fat-2* and *fat-3* mutants convert AA into EPA, *fat-1(wa9) fat-4(wa14)* double mutants cannot [18], [32], [33]. We found that AA or EPA supplementation rescued the sperm guidance defects of *fat-2* mutants, *fat-3* mutants, and *fat-1 fat-4* double mutants (Figure 2, Figure 5, and Figure 6; *P*<0.001). In these experiments, the NA22 bacteria used as a food source do not accumulate PUFAs to high levels (<5% of total membrane lipids), resulting in modest dietary intake [18]. We have not yet developed methods to measure prostaglandins in supplementation experiments. The data, together with previous and published data, strongly support the hypothesis that F-series prostaglandins derived from different precursors function redundantly to promote sperm guidance.

### F-series prostaglandins are not synthesized from D- or E-series intermediates

We next sought to investigate the mechanism by which F-series prostaglandins are synthesized from PUFA precursors. In the mammalian cyclooxygenase-dependent pathway for F2 class prostaglandin synthesis, PGH_2_, PGD_2_, and PGE_2_ are intermediates of PGF_2∝_, 11β PGF_2∝_, and 9β PGF_2∝_ (Figure S1) [22], [29]. To test whether worm extracts contain these intermediates, we used MRM with mass transition *m/z* 351/189, which detects PGD_2_, PGE_2_, and PGH_2_ in the low picomolar range. Several peaks of low abundance were identified in wild-type extracts (Figure 3A). If these peaks represent PGD_2_, PGE_2_, and PGH_2_ isomers, then *fat-1* loss should increase their levels because *fat-1* mutants contain elevated AA and CePGF2. However, this was not the case. The minor peaks observed in wild-type extracts with mass transition *m/z* 351/189 are eliminated in *fat-1* mutants, *fat-4* mutants, and *fat-1 fat-4* double mutants (Figure 3A). This pattern indicates that these compounds likely require EPA, but not AA for synthesis. In addition, CID of parent ions *m/z* 351 at RT = 11.4 min and 12.6 min in wild-type extracts shows similarity to F3 class prostaglandins, with the exception of the minor product ion *m/z* 189 (data not shown). Thus, these compounds are not AA-derived PGD_2_, PGE_2_, or PGH_2_ analogs. These data indicate that worms either do not synthesize PGD_2_, PGE_2_, and PGH_2_ analogs or their concentrations are below the level of detection.

We have previously identified predicted prostaglandin D, E, and F synthases that are required for sperm guidance [20]. *gst-4* and *R11A8.5* encode glutathione S-transferase enzymes related to PGD and PGE synthases, respectively [20]. *gst-4* mutants have more severe sperm guidance defects than the mild defects observed in *R11A8.5* mutants [20]. To test whether these enzymes are essential for F-series prostaglandin synthesis, we conducted LC-MS/MS on *gst-4* and *R11A8.5* null mutant extracts. In four independent trials, we consistently observed a 25–75% reduction in F1, F2, and F3 prostaglandins in *gst-4* null mutants compared to the wild type (Figure S3). *R11A8.5* null mutants had a more complex pattern, with elevated levels of some prostaglandins and reduced levels of others (Figure S3). Null mutations are not currently available for the predicted PGF synthases *Y39G8B.2* and *C35D10.6*. These data indicate that *gst-4* and *R11A8.5* are not essential for F-series prostaglandin synthesis. Instead, they appear to have modulatory roles.

### CePGF2 consists predominantly of ent-PGF_2∝_

Although *C. elegans* lacks cyclooxygenase enzymes, our data strongly support the hypothesis that CePGF2 is synthesized from AA. Furthermore, the CID of CePGF2 is almost identical to PGF_2∝_ (Figure 4). The product ions *m/z* 309 and 193 are characteristic of the F_2∝_ cyclopentane ring [36]. The loss of 44 Da (-C_2_H_4_O) from the parent ion followed by two 18 Da losses (−2× H_2_O), together with the presence of abundant ion *m/z* 193, further indicates the compound has three hydroxyl groups at the C9, C11, and C15 positions (Figure 1B). There is no product ion *m/z* 115 that is abundant in PGF_2∝_ isomers with a hydroxyl group at the C5 position [40]. There are sometimes a few differences in minor product ions of unknown composition between PGF_2∝_ and CePGF2 from wild-type extracts. However, the CePGF2 decomposition pattern from *fat-1* mutant extracts, which have increased CePGF2 concentration, is almost identical to PGF_2∝_ (Figure 4). Minor differences between CePGF2 and PGF_2∝_ spectra could be due to low concentration and co-eluting compounds. We conclude that CePGF2 has chemical composition C_20_H_34_O_5_ with an F_2∝_ cyclopentane ring and hydroxyl groups at positions C9, C11, and C15.

Structural differences in similar compounds often cause changes in chromatographic retention. For example, the RTs of PGF_2∝_ and PGF_3∝_ differ by 34 seconds, although the only structural difference is an additional double bond at C17 in PGF_3∝_ (Figure 1B). Given that CePGF2 and PGF_2∝_ have indistinguishable retention times, we asked whether PGF_2∝_ stereoisomers are resolved in our HPLC program. The C-OH bonds at C9, C11, and C15, the C-C bonds at C8 and C12, and the C = C bonds at C5 and C13 can be in different conformations. We analyzed by LC-MS/MS a series of chemically synthesized PGF_2∝_ stereoisomers that included conformation changes at the C5, C8, C9, C11, and C15 positions. With the exception of the PGF_2∝_ enantiomer (ent- PGF_2∝_), all other stereoisomers were separated (Table 3). The RT differences relative to PGF_2∝_ ranged from 7 seconds for 5-*trans* PGF_2∝_ to 30 seconds for 9β PGF_2∝_. Similar changes were observed with several PGF_1∝_ and PGF_3∝_ stereoisomers in reverse-phase chromatography (Table 3). It is noteworthy that CePGF1, CePGF2, PGF_1∝_, PGF_2∝_, and ent-PGF_2∝_ elute together and the CID patterns of ent-PGF_2∝_ and PGF_2∝_ are identical (Figure 3 and Table 3). These results indicate that CePGF2 is PGF_2∝_, a co-eluting stereoisomer such as ent-PGF_2∝_, or a racemic mixture. To distinguish between these possibilities, we developed a normal phase chiral LC-MS/MS method operated in MRM mode (mass transition 353/193) to separate PGF_2∝_ and ent-PGF_2∝_. When *fat-1(wa9)* mutant extract, which contains abundant CePGF2 (Figure 3), was analyzed by chiral LC-MS/MS, the predominant isomer eluted with the same retention time as the ent-PGF_2∝_ standard (Figure S4). These results strongly support the hypothesis that CePGF2 consists predominantly of ent-PGF_2∝_. Similarly, CePGF1 may be the enatiomer of PGF_1∝_. Nuclear Magnetic Resonance spectroscopic studies will be necessary for absolute confirmation.

### The adult germ line is essential for F-series prostaglandin synthesis

**Figure 7:**
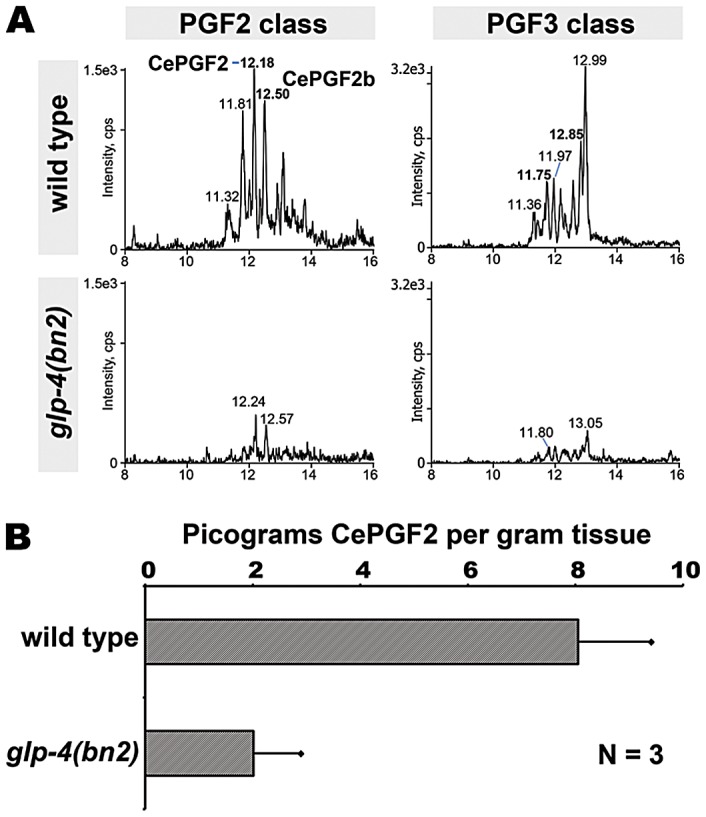
Prostaglandins in adult wild type and germline-deficient *glp-4* mutants. (A) MRM chromatograms of synchronized 1–2 day adult wild-type and *glp-4(bn2)* mutants. The F2 class was detected with mass transition *m/z* 353/193 and the F3 class was detected with mass transition *m/z* 351/193. Liquid chromatography retention time (min) is shown on the X-axis and for selected prostaglandin isomers. Cps, counts per second. (B) CePGF2 quantification in wild-type and *glp-4* mutant extracts. Error bars are SD.

We have previously shown that CePGF2 synthesis requires the RME-2 low-density lipoprotein receptor, which transports PUFAs from the intestine to oocytes [18], [20]. These experiments were conducted with extracts from mixed stage worm cultures. To evaluate the extent to which F-series prostaglandins are synthesized in the adult germ line, we synchronized mass worm cultures from wild-type and *glp-4(bn2)* mutant hermaphrodites. *glp-4(bn2)* is a temperature sensitive mutation that prevents germ cell proliferation and differentiation at the restrictive temperature (25°C) [35]. We extracted prostaglandins from 1–2 day adult wild-type and *glp-4(bn2)* hermaphrodites raised at 25°C. As shown in Figure 7, multiple F2 and F3 class prostaglandins are present in the extracts, including CePGF2 and CePGF2b. The F1 series could not be evaluated because synchronization limits culture size, restricting the number of LC-MS/MS analyses that can be conducted. CePGF2 is less abundant in 1–2 day old adults relative to mixed stage cultures (Figure 3B and Figure 7B). *glp-4(bn2)* mutant adults lacking germ cells and oocytes have severely reduced levels of F2 and F3-series prostaglandins compared to wild-type adults (Figure 7A). CePGF2 was reduced by about 75% (Figure 7B; *P*<0.001). We conclude that the adult germ line is required to synthesize multiple F-series prostaglandins.

### A cytochrome P450 enzyme influences germline prostaglandin metabolism

**Figure 8:**
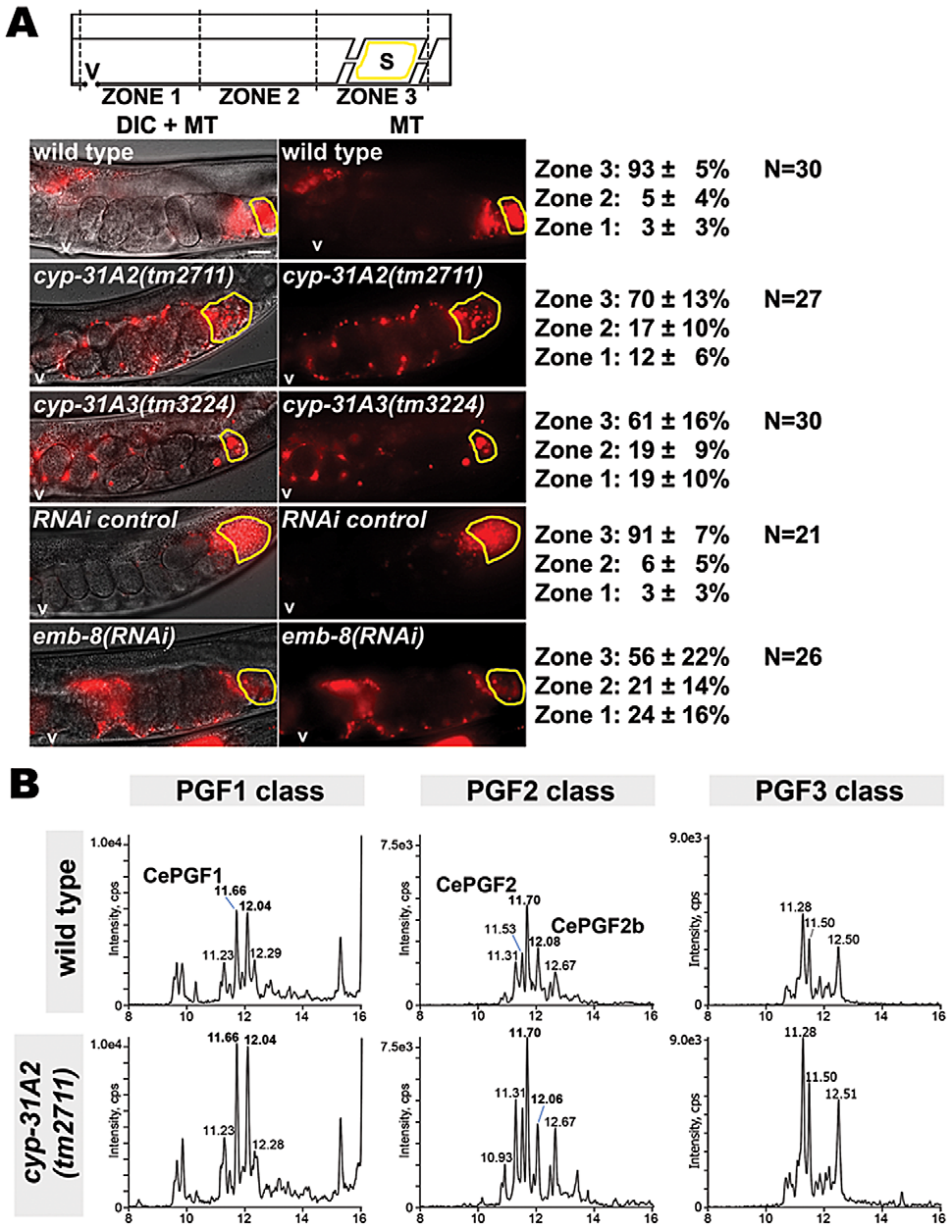
Effect of cytochrome P450 enzymes on sperm guidance and prostaglandin metabolism. (A) Sperm distribution 1 hr after mating wild-type or mutant hermaphrodites to wild-type males. A representative image is shown to the left and average zone distribution ± standard deviation is to the right. The spermatheca (S) is outlined in yellow. V, vulva; MT, MitoTracker channel; N, number of gonads scored. Scale bar, 20 µm. (B) MRM chromatograms of wild-type and *cyp-32A2(tm2711)* mutants. The F1 class was detected with mass transition *m/z* 355/311, the F2 class was detected with mass transition *m/z* 353/193, and the F3 class was detected with mass transition *m/z* 351/191. Liquid chromatography retention time (min) is shown on the X-axis and for selected prostaglandin isomers. Cps, counts per second.

A major remaining question is how F-series prostaglandins are metabolized. Identifying genes required non-autonomously for sperm guidance should provide insight into the pathway(s). In a prior RNAi screen for these genes, we identified *H02I12.8* (also called *cyp-31A2)* [18]. This gene and its paralog *cyp-31A3* encode class 4 cytochrome P450 enzymes most closely related to CYP4V2 [41]. Given that *cyp-31A2* and *cyp-31A3* mRNAs share approximately 92% identity in their coding regions, RNAi might affect both gene products. Previous studies have shown that these two enzymes act redundantly to promote formation of the egg permeability barrier following fertilization [41], [42]. To test whether *cyp-31A2* and *cyp-31A3* are required for sperm guidance, we analyzed their deletion mutants. *cyp-31A2(tm2711)* and *cyp-31A3(tm3224)* single mutants are both fertile with no obvious oogenesis defects. However, wild-type sperm fail to target the spermatheca efficiently in the mutants (Figure 8A; *P*<0.001). Similar results are observed in *emb-8* RNAi hermaphrodites (*P*<0.001). *emb-8* encodes a homolog of NADPH cytochrome P450 reductase, which provides electrons for cytochrome P450s. Sperm velocity in *cyp-31A2(tm2711)* mutants is not significantly different than velocity in the wild type (*P*>0.30), but directional velocity is decreased (Table 1, lines 1 and 9; *P*<0.05). Similar results were previously reported using RNAi [18]. These results indicate that cytochrome P450 function is required for sperm guidance.

To investigate the mechanism, we conducted LC-MS/MS analysis of *cyp-31A2(tm2711)* mutant extracts. MRM in three independent trials showed that F1, F2, and F3 class prostaglandins were consistently elevated in the mutant strain compared to the wild type (Figure 8B). LC-MS analysis in the mass range *m/z* 315–360 did not show global elevation of PUFA-derived compounds, demonstrating specificity for F-series prostaglandins (data not shown). Therefore, *cyp-31A2* is not essential for prostaglandin synthesis. A feature of class 4 cytochrome P450s is the ability to hydroxylate PUFAs and prostaglandins at the C19 and C20 positions [43]. We considered the possibility that *cyp-31A2* is required to form hydroxylated F-series prostaglandins. However, no evidence of these compounds can be been found using LC-MS/MS (Figure S5). MRM with mass transition *m/z* 369/193, which detects 19-hydroxy PGF_2∝_ and 20-hydroxy PGF_2∝_ standards, fails to detect these metabolites in wild-type extracts or *fat-1(wa17)* mutant extracts with elevated CePGF2. Similar results were observed using MRM with mass transition *m/z* 367/193, which detects hydroxylated PGF_3∝_ (Figure S5). We conclude that CYP-31A2 negatively regulates F-series prostaglandin metabolism, likely by affecting a process common to F1, F2, and F3 classes. The increased prostaglandin levels in *cyp-31A2* mutants could be due to continued synthesis in fertilized eggs (see Discussion).

### *C. elegans* and mouse tissues contain similar F-series prostaglandin profiles

**Figure 9:**
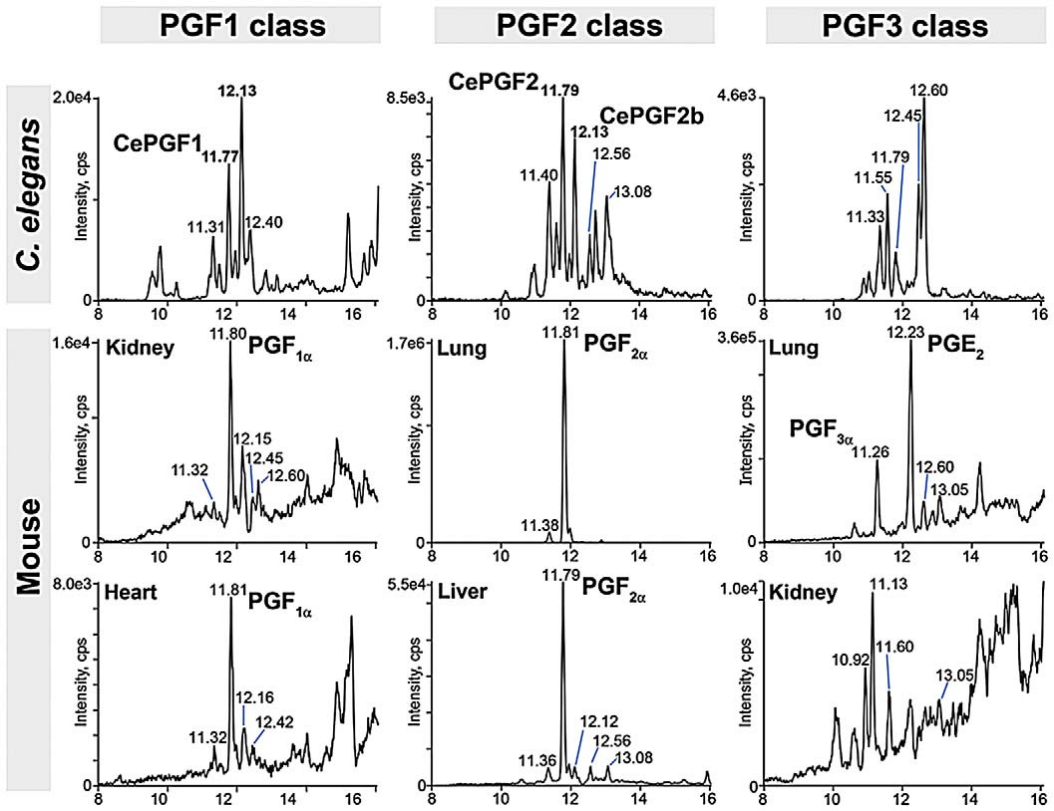
F-series prostaglandins in *C. elegans* and mouse tissues. MRM chromatograms comparing F-series prostaglandins in *C. elegans* and mouse tissue extracts. The F1 class was detected with mass transition *m/z* 355/311, the F2 class was detected with mass transition *m/z* 353/193, and the F3 class was detected with mass transition *m/z* 351/193. Liquid chromatography retention time (min) is shown on the X-axis and for selected prostaglandin isomers. Cps, counts per second. The *C. elegans* MRM chromatograms are from Figure 3A. The mouse lung is extremely rich in PGE_2_, which was detectable with mass transition *m/z* 351/193.

CePGF1 and CePGF2 appear to be identical to or co-eluting stereoisomers of PGF_1∝_ and PGF_2∝_, respectively. However, genetic, biochemical, and pharmacological evidence shows that their biosynthesis is cyclooxygenase-independent [20]. In mammals, PGF_2∝_, ent-PGF_2∝_, and other stereoisomers can be formed independent of cyclooxygenase via free radical-initiated peroxidation [30], [44]. To determine which F-series prostaglandin isomers are synthesized in healthy mammalian tissues, we extracted lipids from adult wild-type mouse liver, heart, kidney, and lung. LC-MS/MS analysis indicates that these extracts contain abundant PGF_1∝_ and PGF_2∝_, which co-elute (Figure 9). The lung is particularly rich in these prostaglandins. On the other hand, PGF_3∝_ could only be detected in lung extracts, perhaps reflecting the low abundance of omega-3 PUFAs in the mouse diet. In *C. elegans*, multiple prostaglandin isomers are synthesized from DGLA and AA independent of cyclooxygenase. We found that mouse tissues also contain putative PGF_1∝_ and PGF_2∝_ isomers, but of relatively low abundance (Figure 9). LC-MS/MS and CID of lung extracts support the view that these compounds are indeed F-series isomers (data not shown). The RTs of these isomers are in many cases identical to the RTs of *C. elegans* isomers. We conclude that extracts from healthy *C. elegans* and mouse tissues contain at least two and perhaps more F-series prostaglandin isomers in common. These results raise the interesting possibility that mammals can synthesize PGF_1∝_ and PGF_2∝_ stereoisomers independent of cyclooxygenase enzymes.

## Discussion

For fertilization to occur, sperm must sense the environment within the female reproductive tract and locate an oocyte that has recently completed meiotic maturation and ovulation. We have been using *C. elegans* to delineate the mechanisms that guide sperm to maturing oocytes [18], [20], [21], [45]. Results from this study and previous studies support the following model. In the intestine, dietary fats are converted into PUFAs and incorporated into yolk lipoprotein complexes [18]. Yolk is secreted into the pseudocoelom and flows to the gonad, where it is endocytosed into oocytes by the RME-2 low-density lipoprotein receptor [34]. Yolk provides oocytes with 20-carbon PUFAs that are converted into at least 10 structurally related F-series prostaglandins, independent of cyclooxygenase enzymes. DGLA, AA, and EPA are major precursors for F1, F2, and F3 classes, respectively, that include PGF_1∝_ and PGF_2∝_ stereoisomers. Conserved glutathione S-transferase and cytochrome P450 enzymes modulate oocyte F-series prostaglandin metabolism, although the biochemical mechanisms are not yet clear.

F-series prostaglandins secreted into the reproductive tract function collectively and partially redundantly to promote sperm velocity and directional motility toward the spermatheca. Prostaglandins provide positional information to sperm that allow them to locate and maintain their place in the spermatheca, despite the obstruction of passing fertilized eggs. As maturing oocytes enter the spermatheca during ovulation, they are immediately fertilized. The CYP-31A2 enzyme acts following fertilization to inhibit prostaglandin synthesis and secretion, ensuring that sperm are not attracted to developing embryos. Endocrine signals such as insulin, as well as signals from somatic gonadal cells transduced by gap junctions regulate oocyte prostaglandin metabolism [20], [21], [45]. Together, these mechanisms help couple nutritional status and oocyte development to reproductive output. New evidence supporting this model is discussed below.

### F-series prostaglandin synthesis from multiple precursors

LC-MS/MS analyses of wild-type and *fat* mutant extracts provide strong evidence that F-series prostaglandins can be synthesized from DGLA, O3AA, AA, and EPA. The concentrations of CePGF1 and CePGF2 in wild-type extracts are similar, although absolute levels of CePGF1 were not determined. The concentrations of F3 class prostaglandins are difficult to estimate because appropriate standards are not available. Given the abundance of EPA in wild-type hermaphrodites and yolk [18], [32], the F3 prostaglandins are likely the most abundant. Precursor levels appear to have a profound impact on prostaglandin synthesis and signaling. For example, *fat-1* mutants have approximately 13-fold higher AA levels relative to the wild type [32], [33]. We found that CePGF2 levels increase by over three fold, helping to compensate for F3 class prostaglandin loss. LC-MS/MS analysis also detected a putative F-series prostaglandin(s) derived from 18:3n3 in *fat-3* mutants. However, F-series prostaglandins derived from 18∶3 or 18∶4 were not identified in wild-type extracts. These results reveal unexpected complexity in the mechanisms that couple PUFA homeostasis to prostaglandin synthesis. A mutant strain completely deficient in prostaglandin synthesis has not been identified. Hence, the extent to which prostaglandins regulate development, immunity, and reproduction is not known.

A key feature of prostaglandin synthesis in *C. elegans* is the absence of cyclooxygenase enzymes [20]. The *C. elegans* genome does not encode cyclooxygenase homologs. Furthermore, drugs that inhibit cyclooxygenase activity do not affect sperm motility [20]. In this study, we show that *C. elegans* extracts contain PGF_1∝_ and PGF_2∝_ stereoisomers, but not key PGH_2_, PGD_2_, and PGE_2_ intermediates of cyclooxygenase-dependent pathways. These data provide compelling evidence that PGF_1∝_ and PGF_2∝_ are synthesized using a cyclooxygenase-independent mechanism. Prostaglandins can be synthesized via a non-enzymatic mechanism mediated by free radical-initiated peroxidation [30]. However, free radical-initiated peroxidation generates a nonselective mixture of prostaglandin stereoisomers, including 8-iso PGF_2∝_ and 8-iso PGE_2_ that are undetectable in *C. elegans*. Free radicals could play a role in worm prostaglandin synthesis, but it seems likely that enzymes are involved. The enantiomeric form of PGF_2∝_, which is not formed by cyclooxygenase and cannot be distinguished from PGF_2∝_ using standard LC-MS/MS or immunoassays, is the major F2 class prostaglandin in human urine [46] and *C. elegans* extracts. An attractive possibility is that F-series prostaglandins can be formed by a conserved, yet undiscovered mechanism that does not involve cyclooxygenases.

### F-series prostaglandins function redundantly

We show that sperm guidance defects depend on the extent to which PUFA classes are collectively eliminated. As each PUFA class with at least three double bonds can serve as a precursor for prostaglandin synthesis, the data support the model F-series prostaglandins function collectively and partially redundantly. Indeed, supplementation of *fat-2(wa17)* mutants, *fat-3(wa22)* mutants, and *fat-1(wa9) fat-4(wa14)* mutants with either AA or EPA largely rescues the sperm guidance defects. Similar results were observed when *fat-2(wa17)* mutants were supplemented with linoleic acid, AA, and EPA and sperm motility was analyzed by time-lapse video microscopy [18]. Microinjecting AA into *fat-2* mutant gonads rescues the sperm distribution defect [18]. Finally, microinjecting nanomolar concentrations of PGF_2α_ or PGF_3α_ into the uteri of *fat-2(wa17)* or *rme-2(b1008)* mutants has an immediate and similar effect on sperm velocity [20]. These results provide strong evidence that F2 or F3 class prostaglandins are sufficient for sperm guidance, yet neither class is essential.

F1 class prostaglandins and prostaglandins derived from 18:3n3 are also likely to function redundantly with F2 and F3 classes. In *fat-1 fat-4* double mutants, the F1 prostaglandins appear to partially compensate for the absence of F2 and F3 classes. Similarly, 18:3n3-derived prostaglandins appear to partially compensate for 20-carbon prostaglandin loss in *fat-3* mutants. Thus, there appears to be tremendous flexibility in the mechanisms that synthesize prostaglandins and transduce their signals. Collective function explains the low abundance of individual prostaglandin isomers in wild-type extracts (relative to mammalian tissues). Why such flexibility and redundancy exists in an animal that generates its own omega-3 and omega-6 PUFAs is not clear, but perhaps it provides a buffer against dietary or genetic changes.

### The cytochrome P450 CYP-31A2 in sperm guidance

CYP-31A2 is a class 4 cytochrome P450 that is most similar to human CYP4V2, which is implicated in the retinal disease Bietti's Crystalline Dystrophy [47]. This enzymatic class has a well characterized function in hydroxylating fatty acids, particularly at the omega-1 and omega-2 positions [43]. Recombinant human CYP4V2 has been shown to hydroxylate EPA and docosahexaenoic acid [48]. In *C. elegans*, RNAi of CYP-31A1/A2/A3 together causes a reduction in hydroxylated EPA metabolites synthesized from microsomes [49]. We were unable to detect hydroxylated forms of F-series prostaglandins in mixed stage wild-type extracts. Thus, potential substrates for CYP-31A2 are EPA and other PUFAs. LC-MS/MS analysis of *cyp-32A2* null mutants indicates that F1, F2, and F3 class prostaglandins are elevated by up to 2-fold. These data support the idea that CYP-32A2 down-regulates prostaglandin synthesis. Time-lapse imaging of sperm motility in *cyp-32A2* null mutants and RNAi hermaphrodites is consistent with a gain of signaling activity, as sperm velocity is unaffected [18]. *cyp-32A2* might act together with or in parallel to *cyp-31A3*, which is also required for sperm guidance.

An interesting possibility is that CYP-31A2 functions to down-regulate prostaglandin synthesis in fertilized eggs. Continued prostaglandin production in embryos might disturb positional information (i.e. the maintenance of a gradient in the uterus), a model supported by sperm guidance data. Consistent with this model, RNAi mosaic studies suggest that CYP-31A2 acts in the germ line, not the somatic gonad [18]. In addition to sperm guidance, CYP-31A2 promotes formation of the permeability barrier in fertilized eggs [42]. This barrier is thought to consist of long chain fatty acids conjugated to sugars and prevents the entry and exit of small molecules (<3,000 Da) [42]. Hence, CYP-31A2 might act after fertilization to divert PUFAs away from prostaglandin synthesis to permeability barrier formation. The permeability barrier could also prevent prostaglandins from being secreted into the extracellular environment.

### Prostaglandins as conserved sperm guidance cues?

Outside of aquatic species, little is known regarding the mechanisms that guide sperm to oocytes. Human sperm are thought to receive multiple signals that guide them to cumulus-enclosed oocytes, which lie in the highly arborized lumen of the oviduct [8], [11], [50]. Recent studies have documented that progesterone and prostaglandins bind to CatSper [9], , a sperm Ca^2+^ channel implicated in hyperactivation and chemotaxis [51], [52]. PGF_1∝_ and PGE_1_ activate CatSper at physiological concentrations, suggesting that these interactions are functional [9], [12]. Although the role of prostaglandins is not entirely clear, they are synthesized by cumulus-oocyte complexes and affect sperm motility *in vitro* [13], [53], [54]. Thus, an attractive scenario is that prostaglandins provide positional information to both human and worm sperm. While there are obvious differences between *C. elegans* and human reproductive systems, it is possible that some molecular features are shared. Delineating shared versus divergent mechanisms should lead to better understanding of those that are fundamental to animal reproduction.

In summary, we show that a heterogeneous mixture of structurally related F-series prostaglandins promotes sperm guidance in *C. elegans*. Prostaglandin synthesis does not involve conventional cyclooxygenase pathways, yet PGF_1∝_ and PGF_2∝_ stereoisomers are still generated. Furthermore, conserved enzymes such as cytochrome P450s regulate prostaglandin synthesis. The *C. elegans* model could provide unanticipated insight into the mechanisms and actions of prostaglandins, which are among the most widespread and important signaling molecules in metazoans.

## Materials and Methods

### *C. elegans* strains, culture, and RNA–mediated interference

*C. elegans* were maintained at 20°C, unless otherwise indicated, and fed with NA22 *E. coli* bacteria, as previously described [18], [20]. The *fog-2(q71)* strain was used for males. The following strains were used: *N2* Bristol (wild type), BX24 [*fat-1(wa9) IV*], BX26 [*fat-2(wa17) IV*], BX30 [*fat-3(wa22) IV*], BX17 [*fat-4(wa14) IV*], BX52 [*fat1(wa9) fat-4(wa14) IV*], SS104 [*glp-4(bn2) I*], *cyp-31A2(tm2711) IV, cyp-31A3(tm3224) IV*, and CB4108 [*fog-2(q71) V*]. The *cyp-31A2(tm2711)* and *cyp-31A3(tm3224)* mutants were provided by the Japanese National Bioresource Project. The *cyp-31A2(tm2711)* strain was backcrossed to the wild type three times. RNAi was performed by the feeding method [55]. HT115 bacterial strains were obtained from the feeding library [56] and sequenced for verification. Wild-type and *glp-4(bn2)* mutant hermaphrodites were grown at 16°C and synchronized to the L1 stage using an egg preparation with minor modifications [57]. The larva were shifted to 25°C and cultured for 2–3 days. For prostaglandin analyses, strains were grown on up to sixty-five 150 mm seeded plates, as previously described [20], [21]. Cultures were supplemented with concentrated bacteria as needed to prevent starvation. Worms were washed off plates with M9 buffer, collected in polypropylene tubes, and stored at −80°C for lipid extraction. Tissue was weighed to ensure that equal amounts were analyzed.

### Sperm guidance assays

MitoTracker Red CMXRos (Invitrogen) was used to stain *fog-2(q71)* males, as previously described [18], [20]. Briefly, about 150 male worms were transferred to a watch glass with 300 µl M9 buffer. Three microliters of a 1 mM MitoTracker CMXRos solution in DMSO was added to the worm solution and mixed. Males were incubated in the dark for 2–3 hours and then transferred to a seeded plate. After 20 minutes, the males were transferred again to a fresh plate and allowed to recover overnight at 16°C. 10–20∼2 day old adult hermaphrodites were anesthetized with 0.1% tricaine and 0.01% tetramisole hydrochloride in M9 buffer for 30 minutes [58]. The anesthetized hermaphrodites were transferred to a plate containing an ∼1 cm drop of bacteria with 50–75 stained males. After 30 minutes of mating, the hermaphrodites were separated from the males and transferred to a fresh seeded plate. To directly observe sperm motility, mated hermaphrodites were mounted immediately onto a 2% agarose pad for time-lapse fluorescence microscopy. DIC and fluorescence images were taken every 30 seconds. Directional velocity toward the spermatheca was measured by creating a straight line through the uterus from the vulva to the spermatheca. The distance traveled along this line from the beginning of a sperm trace to the end was divided by time. Positive values indicate movement toward the spermatheca relative to the starting point. A change in migration direction of greater than 90° within 3 consecutive frames was classified as a reversal. Sperm traces range from a minimum of 2.5 minutes to a maximum of 21 minutes. At least 3 videos from different animals were used for quantification.

To assess sperm distribution, mated hermaphrodites were incubated in the dark for an hour without males and then mounted for microscopy. The reproductive tract was divided into 3 zones, as shown in Figure 1A. Zone 3 was defined as the region spanning the center of the spermatheca plus 50 microns toward the vulva. In cases where large sperm aggregations were adjacent to the spermatheca, zone 3 was expanded to include the entire aggregation. Zones 1 and 2 were defined by measuring the distance from the zone 3 border to the vulva and dividing this region in half. AxioVision software was used to measure distances. A two sample T-test was used to test for significance.

### PUFA supplementation

AA or EPA containing plates were prepared as previously described [18]. Briefly, PUFAs were added slowly to cooled media containing 0.1% NP-40. The final PUFA concentration was 200 µM. Plates were kept in the dark at room temperature for 24 hours and then seeded with NA22 *E. coli* bacteria. Plates were incubated in the dark at room temperature for 3 days. NA22 bacteria accumulate PUFAs in their lipids at ranges from 1–5% [18].

### Lipid extraction

For lipid extraction, 2.0 grams of synchronized adult worms or 6.0 grams of mixed-staged worms were used. Hydrophilic lipids were extracted from frozen worm pellets using a liquid-liquid extraction technique [59]. Briefly, frozen worms were resuspended with 12 ml of ice cold acetone/saline containing 0.005% butylated hydroxytoluene (BHT) to prevent oxidation. Worms were evenly dispersed into twelve 5 ml self-standing plastic tubes for use in the Bullet Blender 5 homogenizer (Next Advance). 0.7–0.8 ml of 0.5 mm diameter Ceria stabilized zirconium oxide beads were added to each tube and the Bullet Blender was set to speed 8 for 2–4 minutes. Homogenization efficiency was checked on a microscope slide. The homogenates were evenly transferred to four 10 ml conical glass tubes. The beads were washed with 4 ml of 1∶2 acetone/saline containing BHT and the wash solution was evenly transferred into the four 10 ml glass tubes. These tubes were centrifuged at 4°C in a clinical centrifuge for 10 minutes at 1000 RCF. The supernatants were transferred to four clean 10 ml conical glass tubes. An equivalent volume of hexane was added to each tube and the tubes were vortexed for 30 seconds. The glass tubes were then centrifuged at 4°C for 10 minutes at 1000 RCF. The upper hexane phase and white debris in the interphase were removed. The lower aqueous phase was acidified to pH 3.5 using 2 M formic acid. An equal volume of chloroform was added to each tube. Next, the tubes were vortexed for 30 seconds and centrifuged at 4°C for 10–15 minutes at 1000 RCF. The lower organic phases from four 10 ml glass tubes were transferred to a clean 15 ml conical tube. The extract was flushed with nitrogen gas and stored at −20°C for at least 24 hours. Next, the aqueous top layer was removed and the organic phase containing prostaglandins was evaporated in a Teflon-lined capped ½-Dram glass vial under a gentle stream of nitrogen gas. The dried lipids were stored at −20°C for up to 2 weeks. For mass spectrometry analysis, the dried extract was dissolved in 200 µl of 80% methanol. For the synchronized adult preps containing 2 grams tissue, the dried extracts were dissolved in 60 µl of 80% methanol to increase prostaglandin concentration.

Mouse heart, lung, kidney, and liver were dissected from eight month old black 6 mice (C57BL/6J). The tissue was immediately frozen in a dry ice bath and stored at −80°C. The tissue weights were 0.50 g heart, 0.41 g lung, 1.04 g kidney, and 1.79 g liver. Liquid/liquid extraction was performed as described above with the following exception: 0.8 ml of 2.0 mm diameter Ceria stabilized zirconium oxide beads and Bullet Blender speed 9 (5 min) were used for homogenization.

### Chromatography and mass spectrometry

LC-MS/MS analyses of commercial standards and tissue extracts were performed as previously described [20], [21] using a system consisting of a Shimadzu Prominence HPLC with a refrigerated auto sampler (Shimadzu Scientific Instruments, Inc., Columbia, MD) and an API 4000 (Applied Biosystems/MDS Sciex, Concord, Ontario, Canada) triple quadrupole mass spectrometer. The chromatographic separation was performed on a Synergy hydro RP-C18 column pre-equilibrated with 0.1% formic acid. The mobile phase consists of 0.1% formic acid [A] and acetonitrile containing 0.1% formic acid [B] and was pumped at a flow rate of 0.2 ml/min. The gradient started with 10% B and went up to 80% B from 0–11 min, 80–100% B from 11–14 min and returned back to 10% B at 16 min. The column effluent was introduced into the mass spectrometer using an ESI interface operating in negative ion mode. Nitrogen was used as a nebulizer and curtain gas (CUR = 10). The collision gas, collision energy and temperature were set at 10, −35 eV and 600°C, respectively. The LC-MS/MS system was controlled by BioAnalyst 1.4.2 software. For comparative analysis of different strains, the extracts and standards were run consecutively.

To determine CePGF2 concentration, a stock solution of PGF_2α_ (1 µg/ml in 80% MeOH) was serially diluted with the same solvent to obtain 1000, 100, 10, 1, 0.1 ng/ml concentrations. The samples were analyzed by the MRM method. The standard curve exhibited excellent linearity in the range of concentration 0.1–1000 ng/ml with a correlation coefficient >0.99. Average CePGF2 concentration was calculated from three MRM analyses using two independent sample extractions.

Separation of PGF_2α_ stereoisomers that co-elute in reverse-phase liquid chromatography was achieved by following a previously published method with modification [46]. Separation was carried out on a ChiralPak AD-H column (4.6 mm×250 mm, 5 m, Chiral Technologies, Exton, PA) with 12.5% ethyl alcohol and 12.5% isopropyl alcohol in hexane containing 0.1% formic acid at 1 ml/min. MRM was carried out in atmospheric pressure chemical ionization (APCI) negative ion mode with mass transition *m/z* 353/193. The instrument conditions were optimized with the collision gas, collision energy and temperature were set at 10, −35 eV and 450°C, respectively.

## Supporting Information

Figure S1 Mammalian F2 class prostaglandin synthesis. (A) Cyclooxygenase-dependent pathways. (B) Structures of arachidonic acid and PGF_2α_.
(TIF)

Figure S2 Prostaglandins in *fat-3* mutant. (A) MRM chromatograms of wild-type and *fat-3(wa22)* mutant extracts. The F1 class was detected with mass transition *m/z* 355/311, the F2 class was detected with mass transition *m/z* 353/193, and the F3 class was detected with mass transition *m/z* 351/193. Liquid chromatography retention time (min) is shown on the X-axis and for major prostaglandin isomers. Cps, counts per second. (B) LC-MS/MS of chemically synthesized PGF_1α_ compared to a putative F-series prostaglandin derived from 18:3n3 in *fat-3* mutant extracts. Red color indicates ions shared by the standard and the unknown prostaglandin, after subtracting the mass difference between DGLA and 18:3n3 (28 Da). Blue color indicates ions that are not shared. *m/z* is on the X-axis. (C) MRM chromatograms of wild-type and *fat-3(wa22)* mutant extracts using the mass transition *m/z* 327/283. 2,3-Dinor-11β-PGF_2α_ is an 18-carbon PGF_2α_ metabolite.
(TIF)

Figure S3 F2 class prostaglandins in *gst-4(ok2358)* and *R11A8.5(ok3316)* mutant extracts. MRM chromatograms using the mass transition *m/z* 353/193. *gst-4* and *R11A8.5* encode glutathione S-transferases with sequence similarities to PGD and PGE synthases, respectively. Liquid chromatography retention time (min) is shown on the X-axis and for major prostaglandin isomers. Cps, counts per second.
(TIF)

Figure S4 The PGF_2∝_ enantiomer co-elutes with the predominant F2 class prostaglandin in *fat-1* mutant extracts using chiral chromatographic separation. Normal phase chiral LC-APCI-MS/MS chromatograms operated in MRM with mass transition *m/z* 353/193. Chromatograms of chemically synthesized standards (top) and mixed staged *fat-1(wa9)* mutant extract (bottom) are shown.
(TIF)

Figure S5 Absence of hydroxylated F-series prostaglandins in wild-type worm extracts. (A) MRM chromatograms of mixed staged *fat-1(wa9)* mutant extracts. The mass transition *m/z* 353/193 was used to detect F2 class prostaglandins and mass transition *m/z* 369/193 was used to detect hydroxylated forms, such as 20-hydroxy PGF_2α_. Liquid chromatography retention time (min) is shown on the X-axis and for major prostaglandin isomers. The retention times for 20-hydroxy PGF_2α_ and 19-hydroxy PGF_2α_ are 9.13 min and 9.19 min, respectively. Cps, counts per second. (B) MRM chromatograms of mixed staged wild-type extracts. The mass transition *m/z* 351/193 was used to detect F3 class prostaglandins and mass transition *m/z* 367/193 was used to detect hydroxylated forms. Cps, counts per second.
(TIF)
