# Supplementary figures and images for: A Heterogeneous Mixture of F-Series Prostaglandins Promotes Sperm Guidance in the Caenorhabditis elegans Reproductive Tract
Source: PLoS Genet. 2013 Jan 31;9(1):e1003271. doi: 10.1371/journal.pgen.1003271 (PMC3561059; doi:10.1371/journal.pgen.1003271)

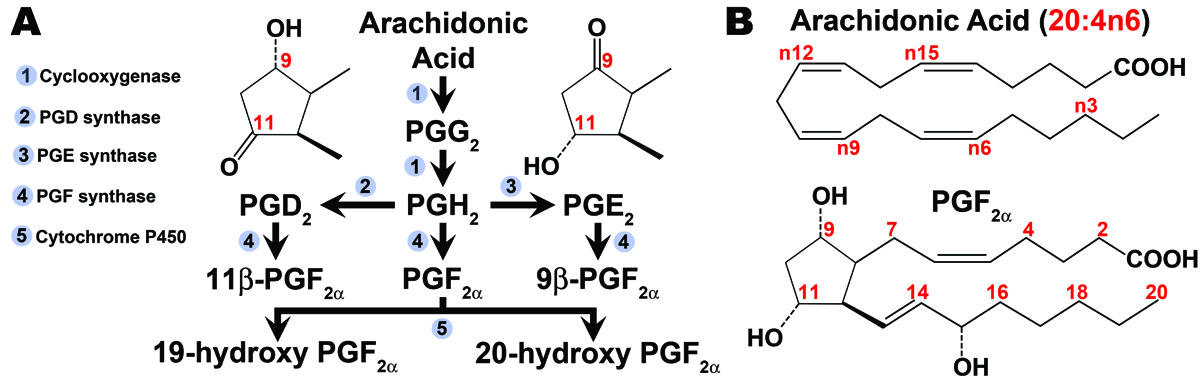

Supplement: Figure S1 — Mammalian F2 class prostaglandin synthesis. (A) Cyclooxygenase-dependent pathways. (B) Structures of arachidonic acid and PGF2α. (TIF) [file pgen.1003271.s001.tif]

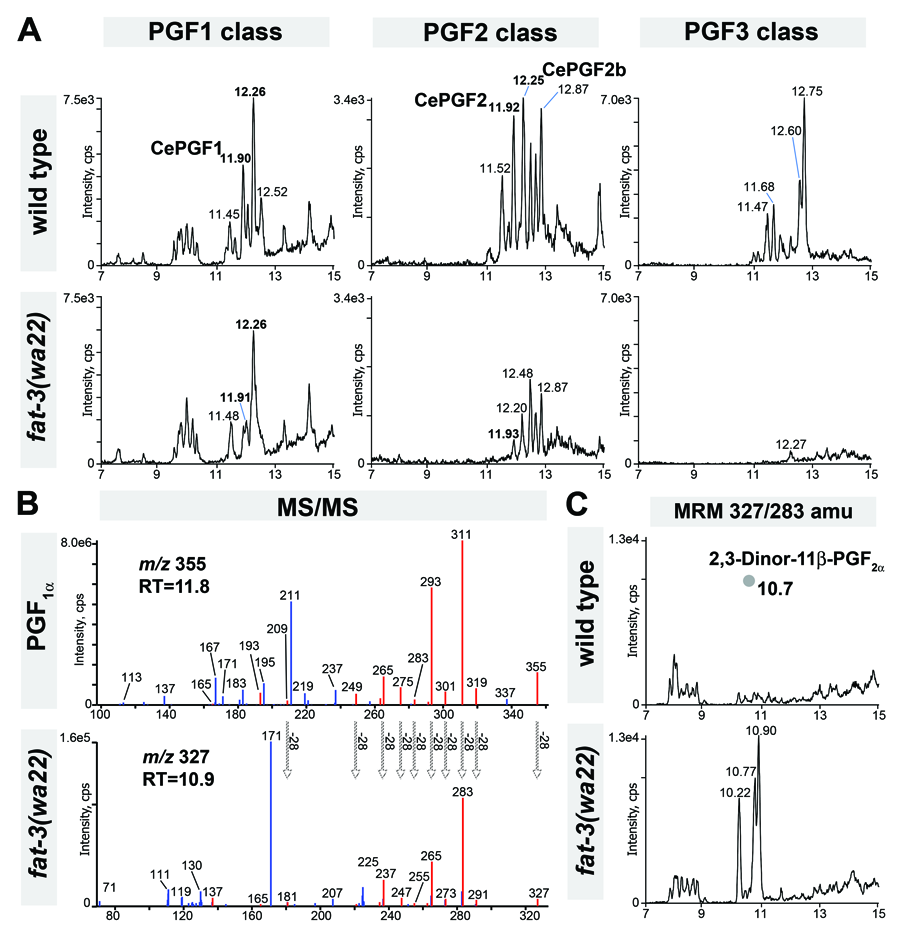

Supplement: Figure S2 — Prostaglandins in fat-3 mutant. (A) MRM chromatograms of wild-type and fat-3(wa22) mutant extracts. The F1 class was detected with mass transition m/z 355/311, the F2 class was detected with mass transition m/z 353/193, and the F3 class was detected with mass transition m/z 351/193. Liquid chromatography retention time (min) is shown on the X-axis and for major prostaglandin isomers. Cps, counts per second. (B) LC-MS/MS of chemically synthesized PGF1α compared to a putative F-series prostaglandin derived from 18:3n3 in fat-3 mutant extracts. Red color indicates ions shared by the standard and the unknown prostaglandin, after subtracting the mass difference between DGLA and 18:3n3 (28 Da). Blue color indicates ions that are not shared. m/z is on the X-axis. (C) MRM chromatograms of wild-type and fat-3(wa22) mutant extracts using the mass transition m/z 327/283. 2,3-Dinor-11β-PGF2α is an 18-carbon PGF2α metabolite. (TIF) [file pgen.1003271.s002.tif]

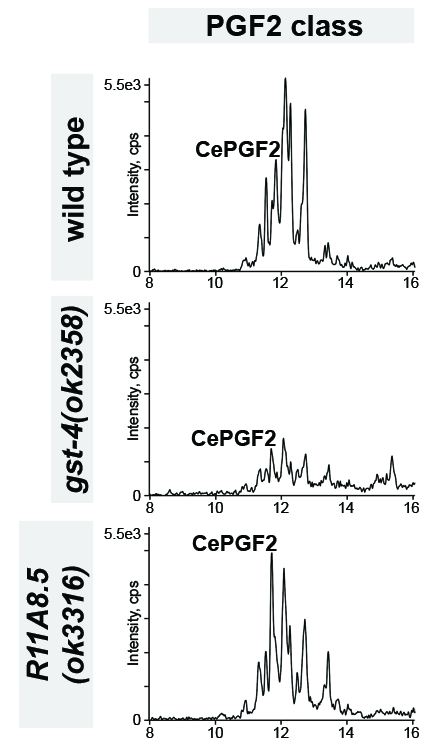

Supplement: Figure S3 — F2 class prostaglandins in gst-4(ok2358) and R11A8.5(ok3316) mutant extracts. MRM chromatograms using the mass transition m/z 353/193. gst-4 and R11A8.5 encode glutathione S-transferases with sequence similarities to PGD and PGE synthases, respectively. Liquid chromatography retention time (min) is shown on the X-axis and for major prostaglandin isomers. Cps, counts per second. (TIF) [file pgen.1003271.s003.tif]

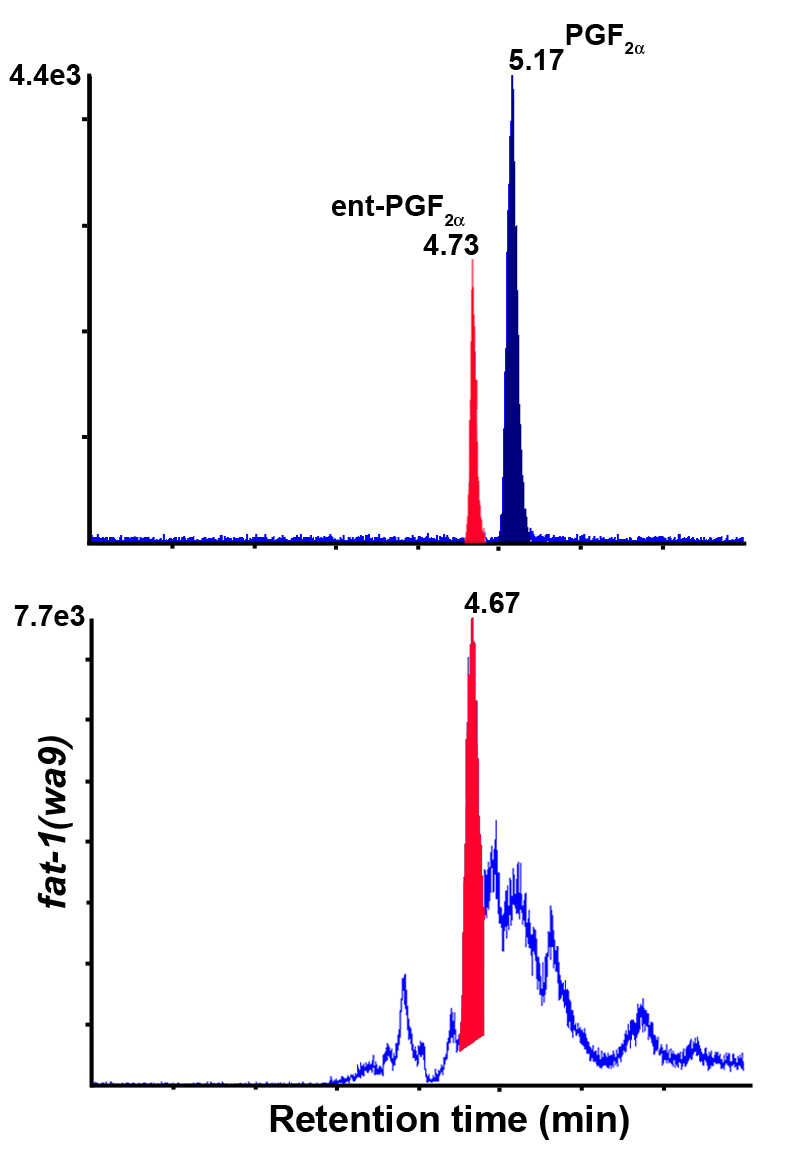

Supplement: Figure S4 — The PGF2∝ enantiomer co-elutes with the predominant F2 class prostaglandin in fat-1 mutant extracts using chiral chromatographic separation. Normal phase chiral LC-APCI-MS/MS chromatograms operated in MRM with mass transition m/z 353/193. Chromatograms of chemically synthesized standards (top) and mixed staged fat-1(wa9) mutant extract (bottom) are shown. (TIF) [file pgen.1003271.s004.tif]

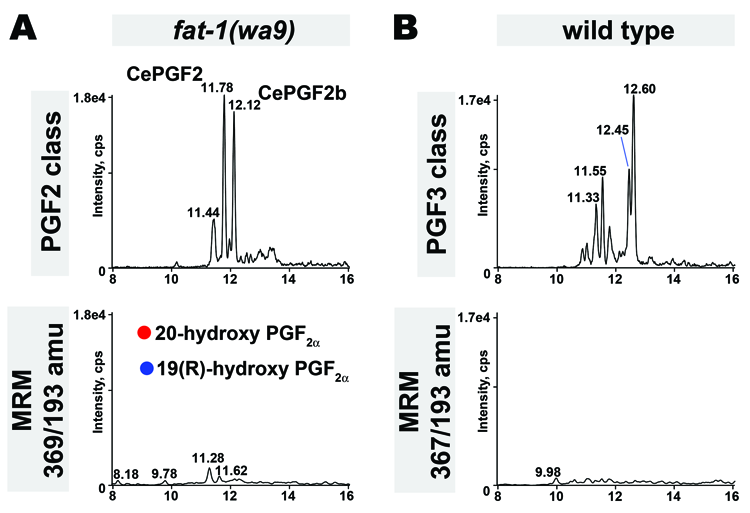

Supplement: Figure S5 — Absence of hydroxylated F-series prostaglandins in wild-type worm extracts. (A) MRM chromatograms of mixed staged fat-1(wa9) mutant extracts. The mass transition m/z 353/193 was used to detect F2 class prostaglandins and mass transition m/z 369/193 was used to detect hydroxylated forms, such as 20-hydroxy PGF2α. Liquid chromatography retention time (min) is shown on the X-axis and for major prostaglandin isomers. The retention times for 20-hydroxy PGF2α and 19-hydroxy PGF2α are 9.13 min and 9.19 min, respectively. Cps, counts per second. (B) MRM chromatograms of mixed staged wild-type extracts. The mass transition m/z 351/193 was used to detect F3 class prostaglandins and mass transition m/z 367/193 was used to detect hydroxylated forms. Cps, counts per second. (TIF) [file pgen.1003271.s005.tif]
